# Comparison of clinical, para-clinical and laboratory findings in survived and deceased patients with COVID-19: diagnostic role of inflammatory indications in determining the severity of illness

**DOI:** 10.1186/s12879-020-05540-3

**Published:** 2020-11-23

**Authors:** Mohsen Rokni, Kazem Ahmadikia, Somaye Asghari, Shahabodin Mashaei, Fahimeh Hassanali

**Affiliations:** 1 Department of Immunology, School of Medicine, Tehran University of Medical Sciences, Tehran, Iran; 2 Department of Medical Parasitology and Mycology, School of Public Health, Tehran University of Medical Sciences, Tehran, Iran; 3 Department of Immunology, Buali Hospital of Laboratory, Zahedan University of Medical Sciences, Zahedan, Iran; 4 Infectious Diseases and Tropical Medicine Research Center, Buali Hospital, Zahedan University of Medical Sciences, Zahedan, Iran; 5 Rajaie Cardiovascular Medical and Research Center, Iran University of Medical Sciences, Tehran, Iran

**Keywords:** COVID-19, SARS-CoV-2, Acute lung injury, Pneumonia, Cytokine storm syndrome, Immune response, Inflammation mediators, ARDS, Ground-glass opacities, Iran

## Abstract

**Background:**

Since December 2019, when a cluster of pneumonia cases due to SARS-CoV-2 initially emerged in Wuhan city and then rapidly spread throughout the world, the necessity for data concerning the clinical and para-clinical features of Iranian patients with COVID-19 was highlighted. Therefore, we aimed to compare the clinical, para-clinical and laboratory evidences of deceased patients with survival group.

**Methods:**

We extracted data regarding 233 patients with laboratory-confirmed COVID-19 from Buali Hospital in Iran; clinical/para-clinical and inflammatory indexes data were collected and analyzed. The data of laboratory examinations and chest CT findings were compared between deceased and survived patients.

**Results:**

The mean age of the patients was 49.8 years and 64% of our patients were male. The acute respiratory distress syndrome occurred in 64 patients, 52 who were admitted to the ICU, which all of them underwent invasive mechanical ventilation, and 28 who died. Lymphopenia (79%), neutrophilia (79%), and thrombocytopenia (21%) were the most frequently observed laboratory findings of the deceased group on admission. Most patients (68%) had a high systematic immune-inflammation (SII) index of > 500 and increased C-reactive protein level (88%). Levels of inflammatory indexes such as neutrophil to lymphocyte ratio (NLR), platelet to lymphocyte ratio (PLR) and SII were documented to be significantly elevated in the deceased group when compared with the patients who survived (*P* < 0.0001, *P* < 0.001, *P* < 0.0001, respectively). The most commonly presented symptoms were fever (70%) and cough (63%) on admission. Headache was uncommon (11%). Ground-glass opacity with consolidation (mixed) was the most common radiologic finding on chest CT (51%). No radiographic or CT abnormality was found in 15 of 204 patients (7%).

**Conclusion:**

Small fraction of patients with COVID-19 may present without fever and abnormal radiologic findings. Elevated NLR, PLR and SII can be considered as prognostic and risk stratifying factor of severe form of disease.

**Supplementary Information:**

The online version contains supplementary material available at 10.1186/s12879-020-05540-3.

## Background

In late December 2019, a cluster of unknown pneumonia cases reported in Wuhan, China. A few days later, the causative agent of this mysterious pneumonia was recognized as a novel betacoronavirus. Subsequently, The causative virus called as severe acute respiratory syndrome coronavirus 2 (SARS-CoV-2) and the relevant disease termed as corona virus disease 2019 (COVID-19) by the World Health Organization (WHO) [1, 2]. At present, the cases of COVID-19 have been detected in many countries around the world. According to the latest data, up to the Aug 30, 2020, the numbers of laboratory-confirmed cases in Iran reached 371,816 of which 21,359 were unfortunately deceased, and 321,421 were recovered. Also 3759 were severe illness. The number of laboratory-confirmed cases in other countries also reached 24,854,140 of which 838,924 were died, according to official data from the WHO.

Many previous studies have shown that COVID-19 patients have significant inflammatory responses, accompanied by a decrease in the absolute count of lymphocytes in the peripheral blood circulation and an enhancement in the number of neutrophil [3]. Mild/moderate form of SARS-CoV-2 infection is the major clinical form of the infection that is characterized by fever, malaise, cough, upper respiratory symptoms, and with or without dyspnea. Therefore, most of these patients do not need hospitalization [4]. However, nearly 15–20% of cases, who show prominent abnormalities in laboratory findings predicting disease deterioration, would develop severe form of the disease which majorly need to be intensively cared and hospitalization in intensive care unit (ICU) [5, 6]. Organ dysfunction including acute respiratory distress syndrome (ARDS), shock, acute cardiac injury and acute renal injury, may occur in 5% of severe cases with COVID-19 [4, 7]. Since, majority of COVID-19 patients show lung involvement on computed tomography (CT) scan imaging patterns, radiological (X ray) examinations have become essential diagnostic method in early detection and assessment of disease progression [8]. Typical chest CT scan findings of COVID-19 include peripherally distributed multifocal ground-glass opacities (GGOs) with patchy consolidations and posterior part or lower lobe involvement predilection [9]. Increasing numbers of pulmonary lesions extent and density of GGOs on CT scan show disease progression [7, 9]. The pathological damages caused by the virus could stimulate a series of inflammatory responses and uncontrolled production of inflammatory cytokines e.g. interleukin (IL)-1, IL-6 and TNF-α, the recruitment of inflammatory macrophages and granulocytes, leading to cytokines storm (CSS), termed as a secondary hemophagocytic lymphohistiocytosis (sHLH) or macrophage activation syndrome, which may follow by disease deterioration, ARDS, septic shock and eventually multiple organ system failure (MOSF) in some patients [10]. Various studies suggested that genes polymorphisms (SNP) of inflammatory interleukin and chemokine are likely associated with genetic susceptibility to SARS-CoV-2, and probably is responsible for developing different form of COVID-19 in different individuals [11, 12].

In this study, we compared the clinical/laboratory findings, imaging manifestation and outcomes of the disease in clinically classified groups including non-severe and severe (consequently death) COVID-19 patients, to find potential prognostic indications contributing to an accurate assessment of the COVID-19 severity.

## Methods

### Design and participants

This cross-sectional study was performed in referral hospital for COVID-19 patients in Zahedan, Iran. Clinical and paraclinical data regarding 233 laboratory confirmed cases of COVID-19 who were hospitalized in our hospital from February 29, 2020 to May 24, 2020 were subjected in the analysis. A verified case of COVID-19 was defined as a positive result on real-time reverse-transcriptase polymerase chain reaction (RT-PCR) assay of naso-pharyngeal or oro-pharyngeal swab specimens. The protocol of the present study was approved by the ethical committee of ZAUMS, and written consent was obtained from the patients or their guardians. The criteria of clinically based classification of our subjects were as follows, mild/moderate (non-severe) form characterizing with the respiratory distress or oxygen saturation ≤ 93%, or severe form if at least one of the following conditions presented: SpO_2_ ≤ 90%, if respiratory failure occurred and mechanical ventilation was required, shock occurred or ICU admission was required.

### Data collection

From all suspected patients with SARS-CoV-2 infection that were admitted to be hospitalized in infectious unit, oro- and naso-pharyngeal swab specimens were collected and stored in virus transport medium (VTM). In addition, fasting venous blood specimen was collected for para-clinical assessment.

For all of the patients, routine blood biochemistry parameters, complete cell blood count (CBC), C-reactive protein (CRP), erythrocyte sedimentation rate (ESR) and chest radiological/CT scan were performed. Routine blood biochemistry analyses included liver function test (LFT), renal function, electrolytes test, lactate dehydrogenase (LDH), myocardial enzymes e.g. creatine phosphokinase (CPK), D-Dimer and status of other virus infection.

### Real-time reverse transcription polymerase chain reaction assay

Viral ribonucleic acid was extracted from the oro- and naso-pharyngeal swab samples using the COVID-19 ORF1ab/N (two regions, so four sequences of primer should be presented) gene nucleic acid detection kit (manual).

Real-time RT-PCR assays for SARS-CoV-2 RNA detection were performed using Quanti Nova Probe RT-PCR Kit (Qiagen) in a Light Cycler 480 Real-Time PCR System (Roche, Basel, Switzerland) as previously described [13]. Each 20 μl reaction mixture contained 10 μl of 2 × Quanti Nova Probe RT-PCR Master Mix, 0.2 μl of QN Probe RT-Mix, 1.6 μl of each 10 μM forward and reverse primer, 0.4 μl of 10 μM probe, 1.2 μl of RNase-free water and 5 μl of RNA as the template. The thermal cycling condition was 10 min at 45 °C for reverse transcription, 5 min at 95 °C for PCR initial activation, and 40 cycles of 94 °C for 10 s and 58 °C for 30 s. According to the cycle threshold (Ct) analysis, if the Ct values of the fluorescein amidites (FAM) channel and victoria (VIC) channel were ≤ 37, and the curve is S-shaped with a significant exponential growth period, the test result specimen regarded as positive, if the Ct value of one channel was ≤ 37, the specimen tested again. If the Ct values of both channels are > 37, and the internal standard channel test result was positive, then the test result specimen regarded as negative.

### Inflammatory indications

Inflammatory indexes were detected using specific parameters of blood analysis. These indexes were as follows: neutrophil to lymphocyte ratio (NLR) by dividing the neutrophil absolute count to the lymphocyte absolute count and platelet to lymphocyte ratio (PLR) by dividing the platelet count to the lymphocyte absolute count were defined. Systematic immune-inflammation index (SII) was calculated as follows: platelet count **×** NLR (per μL).

### Statistics analysis

All of the data presented by mean ± standard error of mean (SEM). Analysis was performed with the IBM SPSS version 23.0 statistical software package, with an independent sample t test for comparison between groups. Descriptive statistics were performed to determine the patient’s diagnostic and clinical features. *P-*value less than 0.05 were indicated statistically significant.

## Results

A total of 1911 patients with non-severe or severe symptoms of COVID-19 during 3 month were admitted in our hospital, of these cases, laboratory diagnosis of 233 patients was confirmed with RT-PCR method. About 12% (28/233) of patients with confirmed diagnosis of this disease were died. Therefore, during the same time, the rate of mortality for all causes in our hospital was 2.04% (39/1911). Of the 233 patients with SARS-CoV-2 infection included in this study, 205 patients were diagnosed as non-severe or severe form of disease (survival group). However, 28 out of 73 who were suffering from severe form of disease, eventually expired (deceased group, [see Table 1]). The mean age of two survival group and deceased group were 47.6 versus 65.3 years, respectively, which was statistically significant different (*P* < 0.0001). Most of the deceased cases (71.4%) and survival cases (62.9%) were male. Also the mean age of the all patients was 49.8 years.

**Table 1:** Demographic features of all patients with and without COVID-19

Clinical features patients COVID-19	Stat	***N*** = 233
Age, years	16–40 Y	77 (33.1%)
	40–60 Y	84 (36.1%)
	> 60 Y	72 (30.8%)
Gender	Male	149 (63.9%)
	Female	84 (36.1%)
Expire		28 (12%)
Intubation	ICU	52 (22%)
	EMS	12 (5.2%)
Recharge		205 (88%)
Severe Pneumonia		73 (31.1%)
Non-severe Pneumonia		160 (68.9%)
**Total Patients Referred**		1911
Total Hospitalization		629
	Transfer to ICU	73
Total RT-PCR Test		1259
	Positive RT-PCR	233
Chest CT-SCAN		3266
**Follow up**	Recurrence after recovery	7
	Death after Recharge	2

### Clinical and laboratory findings

As indicated in Table 2, fever > 38°c (70%) and dry cough (63%) were the main common symptoms on admission. Dyspnea (62%), SpO_2_ < %93 (55%) and muscle (32%) or chest (22%) pain were also observed in some patients. Regarding deceased groups, 71% of the infected patients were men (20 cases) and more than half had underlying diseases (16 patients, 64%), including hypertension (seven, 25%), diabetes (six, 21%), renal and cardiovascular disease (three, 11% [Table 3]).

**Table 2:** Signs and symptoms at admission of COVID-19 patients

Signs and symptoms	Overall (***N*** = 233)
Fever over 38°c	137 (69.6%)
Dry Cough	124 (62.6%)
Dyspnea	123 (62.1%)
Muscle Pain	63 (31.8%)
Chest Pain	30 (21.8%)
Nausea and Vomiting	29 (14.6%)
Chill	24 (12.1%)
Headache	21 (10.6%)
**Saturation O**_**2**_	
SpO_2_ < %93	128 (54.9%)
SpO_2_ > %93	105 (45.1%)

**Table 3:** Baseline characteristics of patients infected with COVID-19

Any Comorbidity	Survival (***N*** = 205)	Deceased (***N*** = 28)
Gender		
Male	129 (62.9%)	20 (71.4%)
Female	76 (37.1%)	8 (28.6%)
Diabetes	32 (15.6%)	6 (21.4%)
Hypertension	30 (14.6%)	7 (25.1%)
Cardiovascular Disease	22 (10.7%)	3 (10.7%)
Pulmonary Disease	12 (5.9%)	5 (17.9%)
Malignancy	6 (2.9%)	1 (3.6%)
Neural System Diseases	4 (2.1%)	1 (3.6%)
Renal Disease	8 (3.9%)	3 (10.7%)
Autoimmunity Disease	2 (1.1%)	2 (7.1%)
Chronic Liver Disease	4 (2.1%)	0

On the admission, lymphopenia (lymphocyte count lower than 1100 μl) (58%), neutrophilia (absolute neutrophil count more than 6300 μl) (25%), and thrombocytopenia (< 125 × 10^9^ per L, 12.4%) were the most principal laboratory abnormalities in our subjects. Regarding survival group, elevated levels of CRP (87.3%), LDH (97.4%), ESR (90.7%), CPK (35%), NLR more than 5 (33.7%), PLR higher than 200 (40.9%), SII index of rather than 500 (64.9%) were recorded. However, in the deceased group, NLR more than 5, PLR higher than 200 and elevated SII were documented in 93, 71.4 and 93% of the patients, respectively. Furthermore, 79% of deceased patients had lymphopenia. Therefore, NLR, PLR and SII indexes were chosen to be statistically analyzed as the most useful prognostic factor for disease deterioration and severity prediction (Table 4).

**Table 4:** Laboratory test of 233 patients with COVID-19

Blood routine (Unit, Normal range)	Total number N (%)	Stat	Total patient ***N*** = 233(%)	Survival ***N*** = 205 (%)	Deceased ***N*** = 28 (%)
Leucocyte (×10^9^/L, range 3.5–9.5)	233 (100%)	Increased	37 (15.9%)	20 (9.8%)	17 (60.7%)
		Decreased	27 (11.6%)	25 (12.2%)	2 (7.1%)
		Normal	169 (72.5%)	160 (78%)	9 (32.2%)
Neutrophil (×10^9^/ L, range 1.8–6.3)	233 (100%)	Increased	58 (24.9%)	36 (17.6%)	22 (78.6%)
		Decreased	17 (7.3%)	16 (7.8%)	1 (3.6%)
		Normal	130 (67.8%)	153 (74.6%)	5 (17.8%)
Lymphocyte (×10^9^/ L, range 1.1–3.2)	233 (100%)	Decreased	132 (57.6%)	110 (53.7%)	22 (78.6%)
		Normal	101 (42.2%)	95 (46.3%)	6 (21.4%)
Platelet (×10^9^/ L, range 125–450)	233 (100%)	Increased	7 (3.1%)	6 (2.9%)	1 (3.6%)
		Decreased	29 (12.4%)	23 (11.2%)	6 (21.4%)
		Normal	197 (84.5%)	176 (85.9%)	21 (75%)
D-Dimer (mg/L; range < 0.55)	34 (15%)	Increased	19 (55.9%)	12 (35.3%)	7 (20.6%)
		Normal	15 (44.1%)	15 (64.7%)	0
ALT (IU/L, range 0–64)	202 (87%)	Increased	22 (10.9%)	19 (10.6%)	3 (13.1%)
		Normal	180 (89.1%)	160 (89.4%)	20 (86.9%)
AST (IU/L, range 8–40)	202 (87%)	Increased	74 (36.6%)	57 (31.8%)	17 (73.9%)
		Normal	128 (63.4%)	122 (68.2%)	6 (26.1%)
ALK.P (IU/L, range 25–320)	195 (84%)	Increased	36 (18.5%)	30 (17.2%)	6 (28.6%)
		Normal	159 (81.5%)	144 (82.8%)	15 (71.4%)
Total Bilirubin (mg/dL, range 0.4–1.3)	180 (77%)	Increased	24 (13.3%)	19 (11.8%)	5 (26.3%)
		Decreased	8 (4.4%)	8 (5.1%)	0
		Normal	148 (82.3%)	134 (83.1%)	14 (73.7%)
Direct Bilirubin (mg/dL, range 0.1–0.3)	180 (77%)	Increased	73 (40.6%)	62 (38.5%)	11 (57.9%)
		Decreased	36 (20%)	33 (20.5%)	3 (15.7%)
		Normal	71 (39.4%)	66 (41%)	5 (26.4%)
Bun (mg/dL, range 5–24)	225 (97%)	Increased	59 (26.2%)	38 (19.1%)	21 (80.8%)
		Normal	166 (73.8%)	161 (80.9%)	5 (19.2%)
Creatinine (mg/dL, range 0.5–1.4)	223 (96%)	Increased	58 (26%)	40 (20.3%)	18 (69.2%)
		Normal	165 (74%)	157 (79.7%)	8 (30.8%)
CPK (IU/L, range 12–160)	183 (79%)	Increased	68 (37.2%)	57 (35%)	11 (55%)
		Normal	115 (62.8%)	106 (65%)	9 (45%)
LDH (IU/L, range 140–280)	173 (74%)	Increased	168 (97.1%)	151 (97.4%)	17 (94%)
		Normal	5 (2.9%)	4 (2.6%)	1 (6%)
C-Reactive Protein (mg/L, 0.0–6.0)	233 (100%)	Increased	206 (88.4%)	179 (87.3%)	27 (96.4%)
		Normal	27 (11.6%)	26 (12.7%)	1 (3.6%)
ESR (mm/h, 2–22)	162 (70%)	Increased	139 (85.8%)	124 (90.7%)	15 (100%)
		Normal	23 (14.2%)	23 (9.3%)	0
NA (mEq/L, 135–145)	209 (90%)	Increased	7 (3.4%)	3 (1.6%)	4 (16%)
		Decreased	96 (45.9%)	85 (46.2%)	11 (44%)
		Normal	106 (50.7%)	96 (52.2%)	10 (40%)
K (mEq/L, 3.5–5.5)	208 (90%)	Increased	3 (1.4%)	0	3 (12%)
		Decreased	49 (23.6%)	43 (23.5%)	6 (24%)
		Normal	156 (75%)	140 (76.5%)	16 (64%)
NLR (index, < 5)	233 (100%)	Increased	95 (40.8%)	69 (33.7%)	26 (92.9%)
		Normal	138 (59.2%)	136 (66.3%)	2 (7.1%)
PLR (index, < 200)	233 (100%)	Increased	104 (44.6%)	84 (40.9%)	20 (71.4%)
		Normal	129 (55.4%)	121 (59.1%)	8 (28.6%)
SII (index, < 500)	233 (100%)	Increased	159 (68.2%)	133 (64.9%)	26 (92.9%)
		Normal	74 (31.8%)	72 (35.1%)	2 (7.1%)

Independent sample t test show that the inflammatory indicator levels were significantly elevated in the patients with non-severe or severe COVID-19 (survival group) when compared with the deceased group (NLR; *P *< 0.0001, PLR; *P *< 0.001, SII; *P *< 0.0001) (Table 5). In addition, serum BUN and creatinine levels in group deceased were significantly increased when compared with the survival group (*P <* 0.0001).

**Table 5:** Comparison of inclusion indicators in the survival and deceased groups COVID-19

Blood routine (Unit, Normal range)	Stat	Total (***N*** = 233)	Mean	±Std. Err. Mean	Sig. (2-tailed)
Leucocyte Count (×10^9^/L, range 3.5–9.5)	Deceased	28	12.3	1.17	0.0001**
	Survival	205	6.1	0.23	
Platelet Count (× 10^9^/ L, range 125–450)	Deceased	28	202.1	18.1	0.480
	Survival	205	215.9	6.56	
Neutrophil Count (×10^9^/ L, range 1.8–6.3)	Deceased	28	11.08	1.12	0.0001**
	Survival	205	4.69	0.27	
Lymphocyte Count (×10^9^/ L, range 1.1–3.2)	Deceased	28	0.79	0.07	0.0001**
	Survival	205	1.14	0.04	
Neutrophil / Lymphocyte Ratio (index, < 5)	Deceased	28	19.28	2.93	0.0001**
	Survival	205	4.96	0.34	
Platelet / Lymphocyte Ratio (index, < 200)	Deceased	28	343.8	66.4	0.001*
	Survival	205	221.7	10.7	
Systematic Inflammatory Index (index, < 500)	Deceased	28	3532.9	565.3	0.0001**
	Survival	205	1163.5	102.9	
BUN (mg/dL, range 5–24)	Deceased	26	51.2	7.18	0.0001**
	Survival	199	18.3	0.65	
Creatinine (mg/dL, range 0.5–1.4)	Deceased	26	2.22	0.28	0.0001**
	Survival	197	1.16	0.02	
C-Reactive Protein (mg/L, 0.0–6.0)	Deceased	28	15.0	0.79	0.151
	Survival	205	13.2	0.44	
SGOT (IU/L, range 8–40)	Deceased	23	80.1	20.2	0.0001**
	Survival	179	40.6	2.43	
SGPT (IU/L, range 0–64)	Deceased	23	48.8	12.1	0.176
	Survival	179	37.4	2.61	
Alkaline Phosphatase (IU/L, range 25–320)	Deceased	21	271.5	17.4	0.339
	Survival	174	253.1	7.76	
Total Bilirubin (mg/dL, range 0.4–1.3)	Deceased	19	1.44	0.31	0.004*
	Survival	161	0.91	0.05	
Direct Bilirubin (mg/dL, range 0.1–0.3)	Deceased	19	0.59	0.21	0.007*
	Survival	161	0.31	0.03	
Creatine Kinase (IU/L, range 12–160)	Deceased	20	438.8	169.3	0.315
	Survival	163	258.1	48.1	
Lactate Dehydrogenase (IU/L, range 140–280)	Deceased	18	820.5	122.1	0.001*
	Survival	155	534.4	26.5	
Sodium (mEq/L, 135–145)	Deceased	25	136.40	1.43	0.698
	Survival	184	136.04	0.28	
Potassium (mEq/L, 3.5–5.5)	Deceased	25	4.2	0.17	0.001*
	Survival	183	3.9	0.03	
ESR (mm/h, 2–22)	Deceased	15	56.1	4.81	0.048*
	Survival	147	46.3	2.17	
D-Dimer (mg/L; range < 0.55)	Deceased	7	1.24	0.06	0.001*
	Survival	27	0.64	0.08	

The values were presented as mean ± SEM. (*P < 0.05, **P ≤ 0.0001)

### Radiography findings

**Fig. 1:**
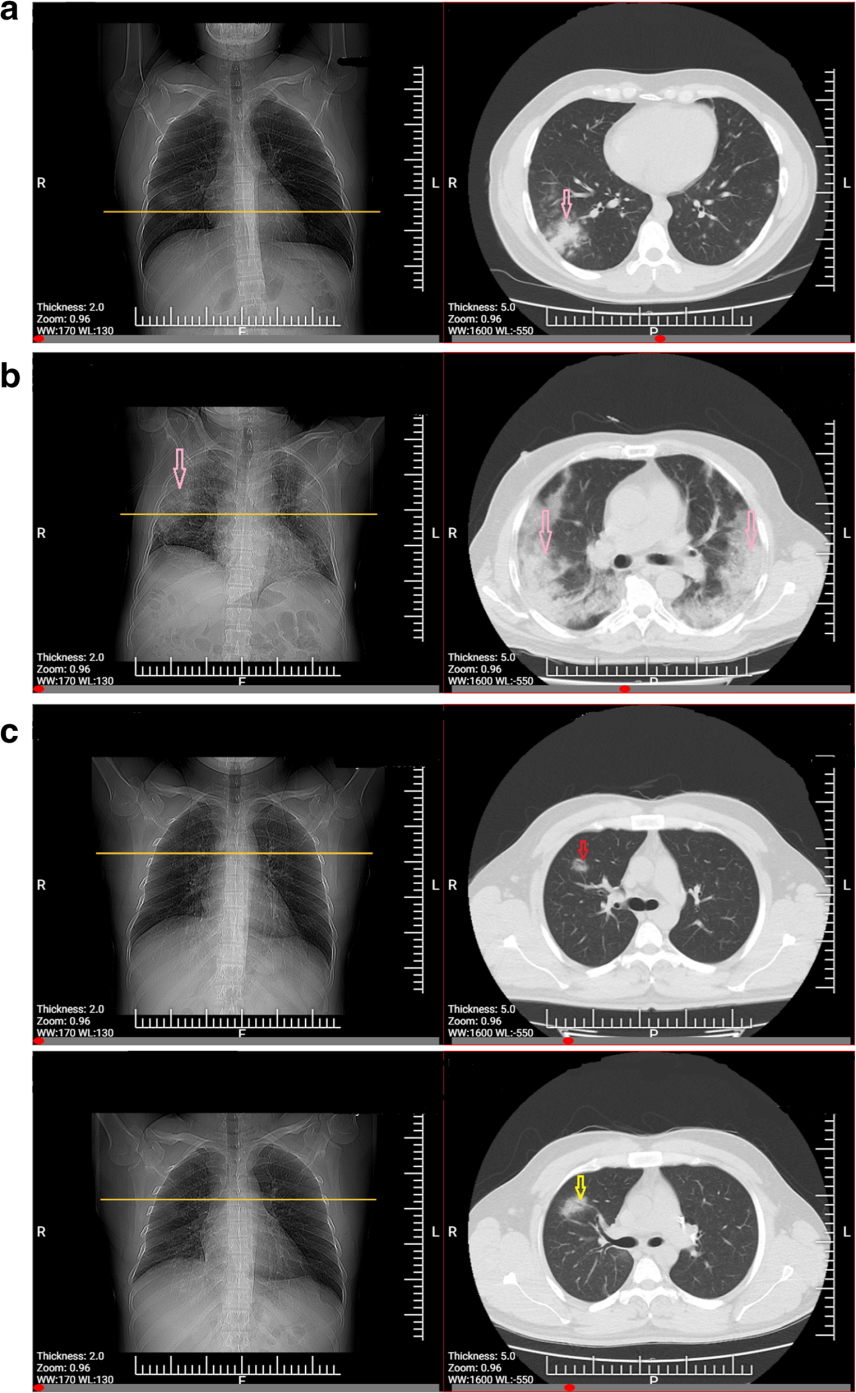
**a**: CT findings of confirmed COVID-19 pneumonia. Consolidation pattern, a young patient presented with fever (38.6 °C) and exposure history. Chest CT acquired on March 28, 2020 showed consolidation lesions (arrow) in the lobe of right lung. **b**: CT findings of severe type confirmed COVID-19 pneumonia. A old patient presented with fever and respiratory occurs and requires mechanical ventilation in ICU. Chest CT was got on the same day as positive RT-PCR with distributed ground-glass opacities with consolidation (arrows) in the lobes of right and left lung. **c**: CT findings of confirmed COVID-19 pneumonia. Ground-glass opacity (GGO) pattern, A young patient presenting without fever with lab-confirmed COVID-19. I: Chest CT acquired 2 days before the first positive RT-PCR test showed a rounded GGO in the right lung upper lobe (arrow). II: Follow-up chest CT-Scan after 3 days showed the size enhancement of the lesion (arrow)

Radiological data is summarized in Table 6. The typical chest CT patterns of patients with proven COVID-19, were GGO pattern (30.3%), patchy consolidations (11.4%, Fig. 1a) and GGO with consolidations (mixed, 51.2% [see Fig. 1b]). Pure GGO lesions (Fig. 1c, I, II) can be the early appearance of SARS-CoV-2 pneumonia. Ground-glass opacity with consolidation (mixed, 89%) was the most frequently observed radiography finding of the deceased group. Interestingly, 15 (7.1%) of participants had clear chest CT scan findings on admission. Except NLR and SII indicators (*P* < 0.01), there was no statically significant difference in indicators of systemic inflammation of patients who had GGO in comparison with those who had mixed pattern on lung CT (Supplementary Figure 1).

**Table 6:** Radiological data of patients with COVID-19

Density Pattern	Survival ***N*** = 176 (%)	Deceased ***N*** = 28 (%)	Lesion Location				Total Count (%)
			No Lesion Count (%)	Right lateral Count (%)	Bilateral Count (%)	Left lateral Count (%)	
Clear	15 (8.5%)	0	15 (7.1%)	0	0	0	**15 (7.1%)**
GGO	63 (35.8%)	0	0	22 (10.5%)	24 (11.5%)	17 (8.3%)	**63 (30.3%)**
Consolidation	20 (11.4%)	3 (10.7%)	0	5 (2.5%)	13 (6.4%)	5 (2.5%)	**23 (11.4%)**
Mixed	78 (44.3%)	25 (89.3%)	0	5 (2.5%)	95 (46.9%)	3 (1.8%)	**103 (51.2%)**
Total	176 (100%)	28 (100%)	15 (7.1%)	32 (15.5%)	132 (64.8%)	25 (12.6%)	**204 (100%)**

## Discussion

Current study is a descriptive report on the clinical/para-clinical characteristics of 233 patients with laboratory proven evidence of COVID-19 attending Buali Hospital, Zahedan, Iran. It represents the recent status of the COVID-19 in east of Iran where biggest state of Iran is located. Collectively, 1911 patients who were suspected to carry the disease were referred to our center over a 3 month period, of which approximately 629 patients had mild/moderate to serious, sometimes fatal, pneumonia and were hospitalized. Given RT-PCR is regarded as reference standard method for diagnosis of SARS-CoV-2 infection [14], the para-clinical data of only 37% (233/629) of patients with positive RT-PCR results were investigated in the present study.

Human coronavirus is one of the main viral pathogens involving respiratory system. SARS-CoV and MERS-CoV besides four other human coronaviruses (HCoV-OC43, HCoV-229E, HCoV-NL63 and HCoV-HKU1) are the main pathogenic viruses belong to coronavirus family causing either severe respiratory syndrome or mild upper respiratory disease [15]. The major SARS-CoV outbreak affected 8422 patients of 29 countries during 2002–2003 [16, 17]. Also, in 2012, MERS-CoV was emerged in Middle East countries [18]. Although, the genomic sequence of SARS-CoV-2 has been shown to be relatively different from the six other coronavirus subtypes but it can be classified as betacoronavirus [15]. In case of SARS-CoV and MERS-CoV, the viruses can be transmitted directly from civets and dromedary camels to humans, respectively, and bats were considered as the origin of both viruses, but the origin of SARS-CoV-2 is still not clear and needs further investigation [15, 19]. Rate of transmission is not exactly documented for SARS-CoV-2; however, human to human transmission has been evidenced [7, 15]. In concert with previous reports, it has been revealed that the clinical manifestations of COVID-19 mimic those presented in SARS-CoV [4, 7, 15, 20]. Fever and cough were the most predominant symptoms manifested in 70% and 63% of our patients, respectively. However, gastrointestinal upsets were infrequently presented (10%), which suggests a different viral tropism and pathogenesis in comparison with SARS-CoV, MERS-CoV, and seasonal influenza [7, 21–23]. The frequency of afebrile patients suffering from COVID-19 (20%) is more frequent than in SARS-CoV (1%) and MERS-CoV infection (2%) [7, 18] implying that presence of fever is not a trustworthy finding to be focused in case definition because afebrile patients will be missed. Similar to previous reports [4, 7, 15], reduced absolute lymphocytes count, and elevated level of CRP and ESR were the main laboratory findings. Damage to T lymphocytes might be a contributing factor leading to substantial decrease in total lymphocytes count and exacerbation of patient’s status [24] as we observed statistically significant abnormalities in laboratory findings (including lymphopenia, elevated NLR, PLR and SII) of patients who expired (deceased group) when compared with survival group. In consistent with our result, a previous study conducted by Liu, et al. [3], documented NLR as the independent risk factor for prediction of severe illness in patients with SARS-CoV-2 infection and should thus have rapid access to an ICU if necessary. So that, in 50% of patients with age ≥ 50 who had NLR ≥ 3.13, severe form of the disease were observed [3, 10]. In another study, a correlation between elevated PLR and the length of hospitalization day was evidenced and it has been concluded that if PLR increased more during treatment, the patient needs longer hospitalization day and had greater possibility of severe pneumonia [25]. Furthermore, Chan et al. in a meta-analysis concluded that NLR and PLR can be served as independent prognostic markers of disease severity in COVID-19 [26]. Recently, NLR and PLR have been validated as prognostic factors in various disorders such as cardiac conditions, solid tumors, sepsis, pneumonia, and ARDS [26]. The SII has been proposed as a prognostic indicator in the follow-up of sepsis patients (Systemic immune inflammatory index in sepsis). In addition, SII has been found to be useful in predicting the prognosis of small cell lung cancer and hepatocellular carcinoma [27, 28]. In consistent with previous study [29], rate of SII was found to be significantly higher in deceased patients when compared with survival group, meaning that it can also be used as predictor of COVID-19 severity and outcome. Therefore, indicators of systemic inflammation such as NLR, PLR and SII may be utilized to predict disease severity, outcome, and mortality of COVID-19. Acute phase proteins such as CRP, LDH, ferritin, procalcitonin, D-Dimer, ESR and IL-6 have also been well correlated with the disease severity, progression and poor outcome in COVID-19 [30]. In our study, CRP protein did not show any significant difference between survival and deceased groups. By the way, pathophysiology of COVID-19 is majorly associated with exaggerated inflammatory responses during the lung involvement. Lymphocytes, especially T lymphocytes, are the main cell to be targeted and consumed by SARS-CoV-2, as does SARS-CoV [24]. Virus particles pass across the respiratory mucosa and attack other cells, resulting in rise of proinflammatory cytokines and stimulate a cytokine storm and a cascade of immune responses in the body, leading to lymphocytic apoptosis, changes in peripheral white blood cells and immune cells such as lymphocytes [15, 31]. In current study, patients who suffered from severe form of COVID-19 (31.3%) required ICU hospitalization and oxygen therapy. Consequently, ARDS and septic shock progressed rapidly in some of our patients, which were eventually followed by multiple organ failure and death. As a result, level of creatinine, BUN and total/direct bilirubin were significantly increased in deceased patients when compared to alive patients which suggesting acute kidney and liver injury in our deceased patients similar to whatever evidenced in former report [32]. Therefore, early diagnosis and promptly treatment initiation of critically ill individuals is issue of crucial importance [15]. Only one patient in our investigation was medical worker. The mortality rate of SARS-CoV and MERS-CoV has been reported as more than 10 and 35%, respectively [33, 34]. The rate of mortality in our SARS-CoV-2 infected population was 12%, resembling to previous study [15]. It necessary to be noted that since patients who had uncomplicated illness and who did not need medical attention were not included in our study, the rate of case fatality in a real world scenario might be even lower. COVID-19 was more commonly observed in men than women (64% vs 36%) in our study. This gender preponderance was in agreement with previous studies [4, 7, 15]. Also, higher rate of MERS-CoV and SARS-CoV infection were documented in males than females [35, 36]. The lower frequency rate of COVID-19, MERS-CoV and SARS-CoV infection in females are thought to be attributed to the protection originating from sex hormones and X chromosome, which play contributing role in innate and adaptive immunity [37]. Additionally, it has been documented that COVID-19 is more probably to occur in older adult males due to weaker immune functions particularly those with chronic underlying diseases [15]. Our patient’s age ranged from 16 to 90 years with a mean age of 49.8 years. The data was in consistent with the previous studies which more and less reported similar mean age [7, 15]. The highest positive rate of COVID-19 RT-PCR was observed in age group 40–60 years. Since, our aim was to investigate the COVID-19 patients with positive RT-PCR as reference method, of 629 hospitalized patients who were clinically suspected cases of COVID-19 and also had initial positive CT scan suggesting COVID-19, only small quantity of our subjects had positive RT-PCR assay suggesting two scenarios: first, it may be indeed be true to say that the sensitivity rate of RT-PCR is as low as 37% which somehow has been also demonstrated in previous studies and may be justified by a list of confounding factors which is regarded to influence the result of RT-PCR and lead to false-negative including: improperly collected, transported or handled specimens, presence of amplification inhibitors in the specimen or inadequate numbers of organisms [14, 38, 39]. Second, due to the overlap of CT imaging patterns between COVID-19 and other viral pneumonia, false-positive cases of COVID-19 may be identified on chest CT scan [14]. Nevertheless, given the rapidly spreading of COVID-19, the priority should be to identify and isolate any suspicious CT scan case in order to administer appropriate treatment. By the way, in the context of disease control, some false-positive cases may be acceptable [14]. Therefore, one negative result of RT-PCR does not rule out SARS-CoV-2 infection and should not be used as the sole basis for patient management decisions and treatment.

## Conclusions

As a result, serially performance of RT-PCR test along with CT scans is recommended. In suspected patients with negative RT-PCR tests, a combination of a history of direct contact with proven cases, clinical manifestations and typical CT imaging features should be collectively used to detect patients with COVID-19. Elevated NLR, PLR and SII can be considered as prognostic and risk stratifying factor of severe form of disease.

## Supplementary Information

**Additional file 1: Supplementary Figure 1.** Relationship between inflammatory indexes and CT scans pattern.

## Data Availability

The datasets used or analyzed during the current study, as well as the study protocol, are available from the corresponding author on reasonable request.

## Abbreviations

COVID-19: Coronavirus disease 2019
SARS-CoV-2: Severe acute respiratory syndrome coronavirus 2
WHO: World health organization
ICU: Intensive care unit
ARDS: Acute respiratory distress syndrome
CT: Computed tomography
GGOs: Ground-glass opacities
IL: Interleukin
CRS: Cytokines release syndrome
SNP: Single nucleotide polymorphism
VTM: Virus transport medium
CRP: C-reactive protein
ESR: Erythrocyte sedimentation rate
LFT: Liver function test
CPK: Creatine phosphokinase
LDH: Lactate dehydrogenase
FAM: Fluorescein amidites
VIC: Victoria
RT-PCR: Real time reverse transcriptase polymerase chain reaction
NLR: Neutrophil to lymphocyte ratio
SII: Systematic immune-inflammation index
PLR: Platelet to lymphocyte ratio
MAS: Macrophage activation syndrome
sHLH: Secondary hemophagocytic lymphohistiocytosis
