# Supplementary figures and images for: Comparison of clinical, para-clinical and laboratory findings in survived and deceased patients with COVID-19: diagnostic role of inflammatory indications in determining the severity of illness
Source: BMC Infect Dis. 2020 Nov 23;20:869. doi: 10.1186/s12879-020-05540-3 (PMC7680983; doi:10.1186/s12879-020-05540-3)

**
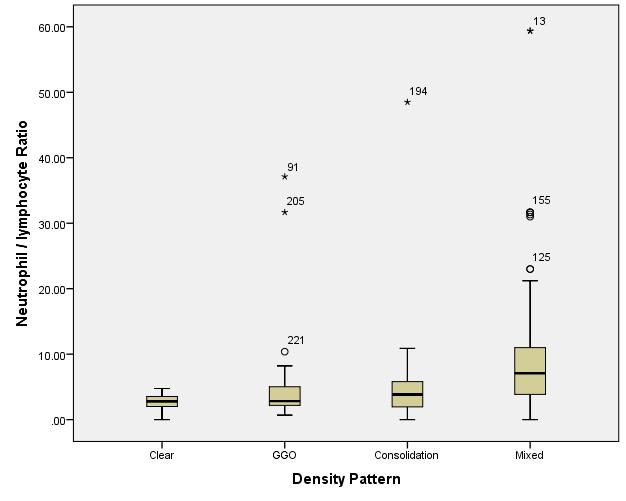

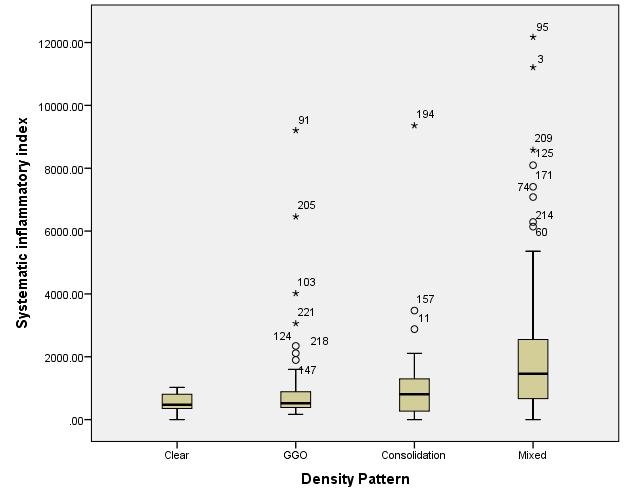

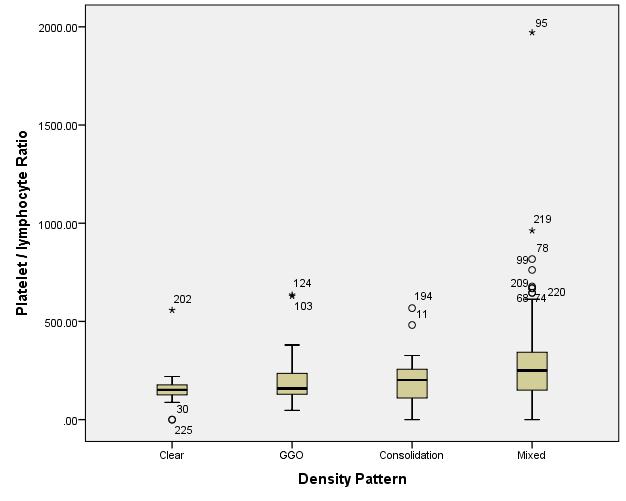
**

**Supplementary Figure 1: Relationship between inflammatory indexes and CT scans pattern**

Supplement: Supplementary file 1 — Additional file 1: Supplementary Figure 1. Relationship between inflammatory indexes and CT scans pattern. [file 12879_2020_5540_MOESM1_ESM.docx]
